# Septic Arthritis and Its Mimics: Assessing the Incidence, Mortality Rate, Cause‐of Death and Preventability in a Population‐Based Cohort Study

**DOI:** 10.1002/hsr2.72845

**Published:** 2026-08-06

**Authors:** Amelia Johansson, Omid Bornai, Jonas Tverring, Oskar Ljungquist

**Affiliations:** ^1^ Division of Infection Medicine, Department of Clinical Sciences, Faculty of Medicine Lund University Lund Sweden; ^2^ Department of Internal Medicine Helsingborg Hospital Helsingborg Sweden; ^3^ Department of Infectious Diseases Helsingborg Hospital Helsingborg Sweden

**Keywords:** acute monoarthritis, incidence, mortality, native knee septic arthritis, preventability

## Abstract

**Background:**

Septic arthritis (SA) is considered the most harmful etiology in patients presenting with an acutely painful swollen knee. This study aimed to estimate the incidence, mortality rate, and cause of death among patients diagnosed with SA and compare it to non‐infectious causes.

**Methods:**

We conducted a population‐based observational cohort study in Skåne, southern Sweden, with a population of 1.4 million people. We included adults in the years 2020 and 2021 with cultures obtained at the emergency department from a native knee, where SA was considered a differential diagnosis based on medical chart review. Data on demographics, comorbidities, microbiological etiology, mortality, and treatment were collected.

**Results:**

Out of 709 knee joint cultures, 60 (8%) cultures were considered SA. The median age of included patients was 71 years (range 19–97) and 67% were men. The incidence of SA was 2.7 cases per 100,000 person‐years. Gout and pyrophosphate arthritis were more prevalent than SA (5.7/100,000 person‐years and 6.1/100,000 person‐years, respectively. The 90‐day all‐cause mortality rate for SA was 8% (4/51), compared to mortality rates of 4% (5/126) for gout arthritis and 4% (5/139) for pyrophosphate arthritis, respectively. Possibly one death within 90‐days was considered directly associated to SA (2%). No deaths were considered preventable due to inadequate antimicrobial therapy or management.

**Conclusion:**

We observed a low incidence of native knee SA, with gout and pyrophosphate arthritis occurring twice as frequently. In our cohort, the infection‐related mortality from SA was low and considered non‐preventable.

## 1 Introduction

Septic arthritis (SA) is defined as a bacterial infection of the joint, most commonly affecting large joints such as the knee or the hip [1]. Differential diagnoses include, crystalloid arthritis (such as gout and pyrophosphate arthritis), rheumatoid arthritis, arthrosis, reactive arthritis and psoriasis arthritis, among others [2]. The incidence of native joint SA in Western Europe has been estimated to 2–10/100,000 person‐years [3]. However, the incidence has a large global variation, with some areas reporting incidences up to 29 cases per 100,000 person‐years [3, 4]. Few studies have previously estimated the incidence of native joint SA in a population‐based manner. SA has previously been associated with short‐term mortality rates between 3% and 11%, in median 39 days from admission to death [5]. However, a nationwide population‐based study from Iceland reports lower short term, in hospital, disease specific mortality rates than previously described, of only 2.7% [6]. Additionally, a French study from 2016 reported a relatively infrequent mortality directly attributable to SA of 5.6%, (median: 24 days [1–42]) [7]. SA has also been associated with significant morbidity, with poor functional joint outcome in almost one‐third of the patients [4, 5, 7, 8]. In 2012, SA resulted in 16,000 emergency department visits in the United States with almost 25% of patients discharged to a skilled facility or with home health [9, 10]. In a US study published in 2019, almost 90% of the 198 deaths of septic patients were considered to be non‐preventable, and only around 10% is potentially preventable [11]. Of the cases with suboptimal care, the most common problems were delay in antibiotics, delay in source control, and inappropriate initial empirical antibiotic treatment relative to final culture results. There is a knowledge gap regarding the clinical impact and outcomes of SA in our clinical setting, particularly when compared to other differential diagnoses. The aim of this study was to estimate the incidence, mortality rate, cause of death, and preventability among patients diagnosed with SA and compare it to non‐infectious causes.

## 2 Methods

### 2.1 Study Design and Setting

This was an observational population‐based cohort study between the years 2020–2021 in Skåne, South Sweden. This region has a population of 1.4 million people, and the Department of Clinical Microbiology, Skåne University Hospital, Lund, is responsible for all microbiological diagnostics in the region. National and regional clinical protocols for SA state that management and treatment should be provided within secondary and tertiary care settings, that is, via emergency departments or inpatient treatment, rather than within primary care. Patient selection followed identical inclusion and exclusion criteria as previously described [12], and we used the same study population to ensure methodological consistency and enable direct outcome comparisons [12]. All cultures obtained from joints registered during the study period were screened for inclusion. Inclusion criteria were adults (≥ 18 years old), and cultures obtained at the emergency department for bacterial culture from a native knee on the clinical suspicion of SA. Patients could be included multiple times in the study.

### 2.2 Microbiology and Data Variables

Medical records were reviewed using the software Melior (Melior, Siemens Healthcare Services, Upplands Väsby, Sweden). Medical students were trained in parallel by a senior infectious diseases specialist (OL) using a study‐specific case report form. Once deemed proficient, they independently reviewed records. Although their entries were not double‐checked, any questions were promptly addressed in consultation with one or both specialists (JT and OL) through consensus. Laboratory Registers at the Department of Clinical Microbiology, Region Skåne, were accessed through LIMS‐systems ww‐lab (Autonik, Nyköping, Sweden). This study was granted ethical approval from the Swedish Ethical Review Authority (DNR‐2021‐05349‐01). All medical records were reviewed according to a predefined study protocol (Supplementary Table S1), including clinical data. Comorbidities were assessed using the Charlson comorbidity score [13, 14].

### 2.3 Case Definition and Other Diagnoses

SA was defined according to a modified version of Newman's criteria, involving patients with an acutely painful and swollen native knee joint, in which analysis from synovial fluid confirmed the presence of a clinically significant bacterial pathogen in culture [15]. Under specific, predefined conditions, the criteria could be applied with exceptions: when antibiotic treatment had been given prior to synovial fluid sampling. In these cases, a diagnosis of SA was still considered possible if two independent reviewers of the medical records, together with a specialist in infectious diseases, were unanimous. Differential diagnoses were also determined through reviews of medical records, using the ICD code and the discharge summary provided by the attending physician as the definitive diagnosis. Mortality rates were expressed as all‐cause mortality and disease‐specific mortality. Preventability was evaluated using a Likert scale. This rating scale ranges from 1 to 6, were 1 is considered to be definitively preventable, and 6 is considered definitively not preventable [11]. Recurrence was defined as a second episode of SA in the same individual, occurring either during or following the step‐down phase of oral antimicrobial therapy. Data related to the management of SA were also collected. Both manual joint flushing via arthrocentesis and arthroscopic lavage were categorized under “joint rinse.” Patients who endured arthroscopic synovectomy, arthrotomy, or other surgical interventions were arranged in the “joint surgery” group. Polymerase chain reaction (PCR) analysis of synovial fluid was not considered in this study; therefore, bacterial pathogens not detectable through conventional culture methods—such as *Borrelia burgdorferi*—could not be identified as a responsible agent. Bacteria that belong to the microbiota of the skin, such as coagulase‐negative staphylococci (CoNS, other than *S. lugdunensis*), *Corynebacterium, Cutibacterium* etc., were not considered relevant organisms, as they are highly unlikely to cause SA of the native knee. Accordingly, we predefined a list of pathogens that were considered contaminants. (Supplementary Table S2). Gonococcal arthritis is often considered a distinct clinical entity with a different course and prognosis and are therefore excluded from this study.

### 2.4 Statistical Analysis

Normally distributed variables were reported as means and were tested with student *t*‐test and nonnormally distributed variables were reported as medians and tested using Kruskal–Wallis test for continues variables. Categorical variables were tested using Fisher's exact test, anda *p*‐value < 0.05 was considered significant. *p* values and confidence intervals (CI) in descriptive statistics were calculated using PRISM (GraphPad, San Diego, United States). Incidences were calculated by dividing annual cases of SA with the annual population. The incidence data were age standardized according to the European standard population.

### 2.5 Ethical Considerations

This study was approved by the Swedish Ethical Review Authority (DNR‐2021‐05349‐01). The need for informed consent was waived by the committee.

## 3 Results

### 3.1 Patient Characteristics

**Figure 1:**
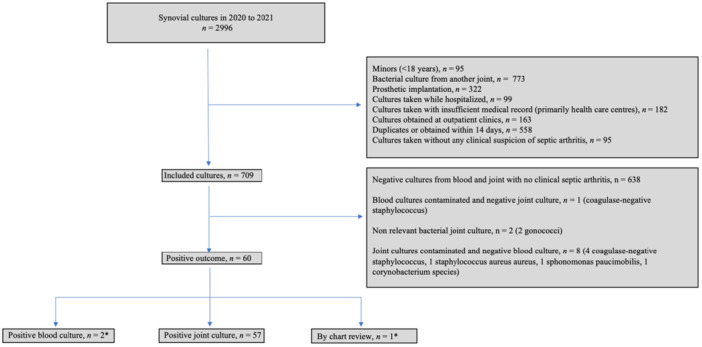
Flow chart of participants. *Two cases were considered positive outcomes despite negative synovial cultures, according to the medical chart review. One case had *Streptococcus agalactiae* detected in blood cultures, while the other had been treated with piperacillin/tazobactam before the cultures were taken. Another case was considered as positive outcome despite negative cultures by medical chart review.

In total, 2996 joint cultures were sent to the department of Clinical Microbiology during 2020 and 2021. A total of 709 bacterial cultures obtained from native knee joints in 668 patients were included in this study (Figure 1). The most common reasons for exclusion were culture doublets, followed by cultures taken within primary health care. The median age for all the patients included in this study was 71 years (range 19–97) and 67% (*n* = 447) were men. The most common comorbidities were diabetes mellitus (17%), previous myocardial infarction (11%) and stroke (11%). The mean score of the Charlson comorbidity index score were 1 point (range 0–13) (Table 1).

**Table 1:** Baseline characteristics of study population.

Baseline characteristics	Study population *n* = 668 patients	Septic arthritis *n* = 51 patients	Pyrophosphate arthritis *n* = 132	Gout *n* = 122	Others *n* = 363
Age, years median (range)	71 (19–97)	72 (25–95)	80 (26–97)	74 (35–94)	68 (18–97)
Male sex (%)	447 (67)	37 (73)	90 (68)	106 (87)	214 (59)
Female sex (%)	221 (33)	14 (27)	42 (32)	16 (13)	149 (41)
Myocardial infarction (%)	72 (11)	9 (18)	20 (15)	16 (13)	27 (7)
Cardiac heart failure (%)	69 (10)	7 (14)	16 (12)	23 (17)	23 (6)
Peripheral vascular disease (%)	40 (6)	7 (14)	11 (8)	8 (7)	14 (4)
Stroke (%)	74 (11)	4 (8)	22 (17)	21 (17)	27 (7)
Dementia (%)	42 (6)	1 (2)	15 (11)	12 (10)	14 (4)
Chronic obstructive pulmonary disease (%)	45 (7)	5 (10)	9 (7)	16 (13)	15 (4)
Connective tissue disease (%)	62 (9)	6 (12)	8 (6)	7 (6)	41 (11)
Chronic liver failure (%)					
Mild (%)	18 (3)	2 (4)	5 (4)	3 (2)	8 (2)
Moderate to severe (%)	1 (0)	0 (0)	1 (1)	0 (0)	0 (0)
Diabetes (%)					
Uncomplicated	101 (15)	9 (18)	34 (24)	19 (16)	39 (11)
End organ damage	14 (2)	4 (8)	4 (3)	3 (2)	3 (1)
Hemiplegia (%)	5 (1)	1 (2)	1 (1)	2 (2)	1 (0)
Chronic kidney disease (%)	60 (9)	8 (16)	12 (9)	18 (15)	22 (6)
Tumor (%)					
Localized (%)	28 (4)	0 (0)	8 (6)	9 (7)	11 (3)
Metastatic (%)	12 (2)	1 (2)	5 (4)	0 (0)	6 (2)
Leukemia (%)	14 (2)	2 (4)	6 (5)	1 (1)	5 (1)
Lymphoma (%)	6 (1)	0 (0)	2 (2)	1 (1)	3 (1)
Acquired immunodeficiency syndrome (%)	0 (0)	0 (0)	0 (0)	0 (0)	0 (0)
Charlson comorbidity index, median (range)^a^	1 (0–13)	1 (0–7)	1 (0–13)	1 (0–9)	0 (0–8)
Urine catheter (%)	44 (7)	10 (20)	15 (11)	8 (7)	11 (3)
Drug abuse (%)					
Alcohol (%)	32 (5)	3 (6)	8 (6)	15 (12)	6 (2)
Intravenous drugs (%)	5 (1)	1 (2)	0 (0)	0 (0)	4 (1)
Smoker (%)					
No smoker (%)	450 (67)	29 (59)	91 (69)	76 (62)	254 (70)
Active smoker (%)	72 (11)	7 (14)	13 (10)	11 (9)	41 (11)
Previous smoker (%)	146 (22)	15 (29)	28 (21)	35 (29)	68 (19)
Rheumatoid arthritis (%)	31 (5)	6 (12)	1 (1)	1 (1)	23 (6)
Immunosuppression^b^ (%)	89 (13)	12 (24)	22 (17)	8 (7)	47 (13)

^a^ Charlson comorbidity index were calculated excluding age.

^b^ Baseline characteristics for the included population. Immunosuppression was defined as a daily corticosteroid intake, chemotherapy, or other medication with immunosuppressive effect, such as TNF‐alpha inhibitors. Immunosuppressive hematologic disorders were also included in this category.

### 3.2 Diagnoses and Incidences

**Figure 2:**
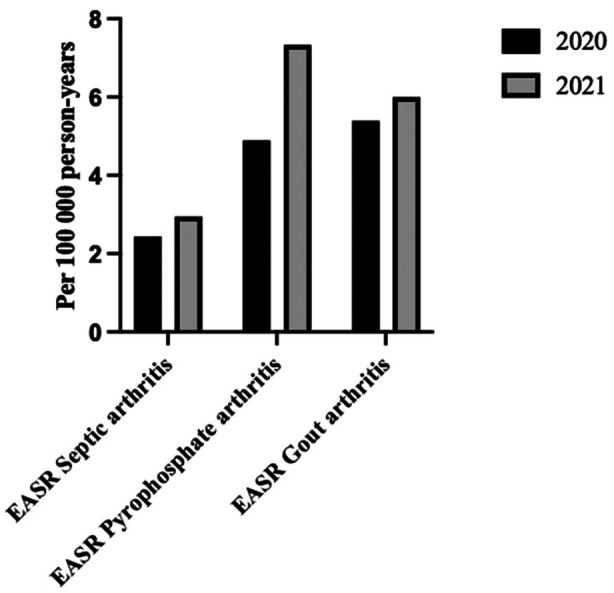
Age adjusted incidences of septic arthritis, pyrophosphate arthritis, and gout arthritis.

Out of 709 bacterial cultures during the study period, 60 cases (8%) in 51 patients were considered SA (Figure 1). The most common diagnoses were pyrophosphate arthritis *n* = 132 (20%) and urate crystallization *n* = 122 (18%). Other diagnoses are specified in Supplementary Table S3). The mean incidence for native knee SA in Skåne during 2020 and 2021 was 2.7/100,000 person‐years. The mean incidence of pyrophosphate arthritis of the knee was 6.1/100,000 person‐years, and the mean incidence of gout arthritis was 5.7/100,000 person‐years (Figure 2). The length of hospitalization was considerably longer for SA patients (median 16 days vs. 2 and 0 days, respectively) compared to pyrophosphate arthritis and gout. Out of the 60 patients with SA, three patients (5%) were admitted due to relapse during our study period. One patient experienced two relapses, both after discontinuing oral antibiotics. Two other patients experienced relapses of SA, one during oral antibiotics and one after discontinuing oral antibiotics.

### 3.3 Thirty‐ and 90‐day Mortality Rates and Preventability

There were no major differences in 30‐day all‐cause mortality rates between SA (3%, *n* = 2), pyrophosphate arthritis (1%, *n* = 1), or gout (2%, *n* = 2). At 90 days, SA demonstrated the highest all‐cause mortality rate (8%) compared to pyrophosphate arthritis (4%, *n* = 5) and gout arthritis (4%, *n* = 5), respectively, although this difference was not statistically significant (*p* = 0.32) (Table S4). Only one death within 90 days (2%, *n* = 1) were possibly considered directly attributed to the knee pathology itself, with the majority of deaths resulting from severe underlying diseases (Table 2). All five patients with SA who died within 90 days suffered from several comorbidities (median Charlson comorbidity index 7, range 4–8). They all received intravenous empiric antimicrobial treatment, and four out of five patients received intravenous antibiotics within 3 h and one patient within 6 h. Four deaths were considered non‐preventable, corresponding a six in the preventability Likert scale [11]. One fatality corresponded to a five on the scale, moderately unlikely to be preventable. This patient received empirical broad spectrum antimicrobial treatment and bedside joint rinse. Later, the patient deteriorated and concurrently received treatment för Herpes simplex encephalitis. The cause of death was considered to be due to multiorgan failure, however the respective contributions of SA and concomitant severe infection are uncertain since herpes encephalitis itself is associated with high mortality. When perusing the causes of deaths for patients with gout and pyrophosphate arthritis, both the short‐ and long‐term deaths were due to serious underlying health conditions, and a few unknown causes of death, corresponding to a six in the preventability Likert scale [11].

**Table 2:** Case summary of patients that succumbed.

Case summary	Underlying cause of death	Likert scale preventability	Case description^a^
90‐day mortality septic arthritis (5 patients, 8%)			
Patient 1 (died within 30 days)	Septic arthritis, herpes encephalitis	5	This patient received broad spectrum antimicrobial treatment and bedside joint rinse. Later, the patient deteriorated and received treatment för Herpes encephalitis. The cause of death was considered to be due to multiorgan failure, however the respective contributions of septic arthritis and concomitant severe infection are uncertain since herpes encephalitis itself is associated with high mortality.
Patient 2 (died within 30 days)	Duodenal ulcers	5	The patient suffered from sepsis as a result from septic arthritis, and duodenal ulcers when admitted to the hospital. The patient died as a result from secondary multiple duodenal ulcers and passed away as a result of bleeding.
Patient 3 (died within 90 days)	Infection with Covid‐19.	6	The patient was discharged after 17 days to a nursing home with oral antibiotics. The patient returned to the hospital after 14 days with dyspnea, fever and impaired general condition, positive for covid‐19, which was regarded the cause of death.
Patient 4 (died within 90 days)	Kidney failure.	6	The patient received palliative care due to severe comorbidities during the hospital stay and was discharged to a nursing home. After 3 days the patient returned to the hospital with loss of consciousness, kidney failure and CRP of 242. The patient received only palliative care and passed away.
Patient 5 (died within 90 days)	Unknown cause of death.	6	The patient passed away outside the hospital and therefore the cause of death is unknown.
90‐day mortality pyrophosphate arthritis (5 patient, 4%)			
Patient 1 (died within 30 days)	Kidney failure.	6	The patient died as a result of severe kidney failure
Patient 2 (died within 90 days)	Unknown cause of death.	6	The patient died outside the hospital (unknown death).
Patient 3 (died within 90 days)	Myelofibrosis.	6	The patient died as a result from worsening of myelofibrosis.
Patient 4 (died within 90 days)	Urosepsis.	6	The patient died due to sepsis from the urinary tract.
Patient 5 (died within 90 days)	Critical ischemia.	6	The patient passed away as a result from critical ischemia of the leg.
90‐day mortality gout arthritis (5 patients, 4%)			
Patient 1	Unknown death.	6	The patient died outside the hospital (unknown death).
Patient 2	Intracranial bleeding.	6	The patient suffered from severe intracranial bleeding due to blood thinners (warfarin) and passed away.
Patient 3	Pancreatic cancer.	6	The patient was diagnosed with metastatic pancreas tumor during the hospital stay and later died due to secondary bowl infection and pneumonia.
Patient 4	Unknown death.	6	The patient died outside the hospital (unknown death).
Patient 5	Unknown death.	6	The patient died outside the hospital (unknown death).

^a^ The ratings from 1 to 6 correspond to a Likert scale assessing preventability, ranging from 1 (definitely preventable) to 6 (definitely not preventable due to a rapidly fatal condition at admission and/or predefined care limitations). Intermediate values reflect varying degrees of likelihood that the event could have been prevented under optimal circumstances, where 5 is considered moderately likely not to be preventable.

## 4 Discussion

In this 2 year population based cohort study in southern Sweden, the mean incidence of native knee SA was low (2.7/100,000 person‐years) with the differential diagnoses gout and pyrophosphate arthritis more than twice as common (5.7/100,000 person‐years and 6.1/100,000 person‐years, respectively). Empirical antimicrobial treatment was common, with 30%–47% of all non‐SA diagnoses receiving intravenous antimicrobial treatment therapy depending on diagnosis. The all‐cause and disease‐specific 30‐ and 90‐day mortality rates for SA were in the lower range compared to previous published estimates, and no deaths were considered preventable due to inadequate antimicrobial therapy or management. In both the pyrophosphate and gout cohorts, all recorded deaths were attributed to comorbid conditions rather than to the underlying joint disease itself.

### 4.1 Incidence

Previous studies estimated the incidence of native joint SA to 2–10/100,000 person‐years, and our results are in the lower range [3]. A recent Swedish study estimated the incidence of SA in adults at 4 per 100,000 person‐years, slightly higher than our findings. However, this study relied solely on ICD‐10 codes without microbiological confirmation, which might explain the discrepancy [16]. Several factors likely contributed to our lower estimates: our study period encompassed the COVID‐19 pandemic, when healthcare‐seeking behavior was reduced, we applied stricter diagnostic criteria requiring microbiological or synovial fluid confirmation (disregarding contaminants), and our analysis was specifically limited to native knee SA rather than all joints.

Like previously reported findings, our study found that SA was more prevalent in men than in women. Gout and pyrophosphate arthritis were more than twice as common compared to SA in patients presenting to the ER with a monoarthritis of the knee in our study. The true incidences of these diagnoses in our region might have been higher than what we found in this study, as cases could theoretically have been managed in primary healthcare, and we only considered emergency department visits. Other studies states that the annual incidence of gout show variation where incidence rates vary between 58 and 289/100,000 person‐years [17]. A recent retrospective observational study including all joints and prosthetic joints in outpatients and inpatients from the United States, estimated the incidence of acute pyrophosphate arthritis to 1140/100,000 person‐years [18]. This is much higher than our incidences and may partly be a result from differences in case finding. In our study, we included only patients that underwent arthrocentesis at the emergency departments. The low incidence in our setting may also be due to the Covid‐19 pandemic, since people did not seek health care to the same extent during the pandemic.

### 4.2 Outcome

SA had the highest 90‐day all‐cause mortality rate of 8%, compared to pyrophosphate arthritis (4%) and gout arthritis (4%). However, when we analyzed the cause of death in the patients with SA, all except one of these patients died with SA rather than because of the SA. The observed deaths appear to reflect an elder, comorbid population, rather than the disease specific mortality. The disease specific 90‐day mortality rate of SA was low at 2%. SA has previously been associated with mortality rates as high as 15% [5], however, later studies have reported lower mortality rates more in line with our results; two European studies from 2008 to 2016 reports mortality rates as low as 2.7% and 5.6%, respectively [6, 7]. The shift towards lower mortality rates in patients with SA might be owing to modern healthcare, including enhanced diagnostic capabilities and more timely therapeutic interventions. SA has traditionally been regarded as a severe condition associated with significant mortality, leading to frequent antibiotic administration in patients presenting with monoarthritis in emergency settings [4]. In our cohort, the incidence of SA was 8%, and the 30‐day mortality was 2%. This is much lower than other severe acute infections, such as bloodstream infection, with short‐term mortality rates up to 30% [19]. Despite this, nearly 40% of patients with other diagnoses than SA received antimicrobial therapy. These findings highlight a potential opportunity to improve antimicrobial stewardship, particularly in the context of rising global antimicrobial resistance. Furthermore, an exclusive clinical focus on SA may contribute to delayed recognition and treatment of crystal arthropathies, potentially leading to suboptimal patient outcomes and unnecessary antibiotic exposure.

### 4.3 Preventability

In our cohort, no deaths in the SA group were classified as preventable, which likely reflects the high attention to early antimicrobial therapy and the impact of significant underlying comorbidities in these patients. Similarly, no deaths in the pyrophosphate or gout arthritis groups were considered preventable, as severe pre‐existing health conditions were consistently identified as contributing factors upon further analysis. This suggests that the observed mortality rates for patients in this cohort, regardless of diagnosis, be primarily attributable to serious underlying comorbidities rather than the direct effects of these diseases themselves. Consequently, the mortality rates observed in these groups likely align with the general mortality rates of this patient population, although this was outside of the scope of the current investigation.

### 4.4 Strengths and Limitations

A key strength of this study is its population‐based design, which included all patients meeting our inclusion criteria from 2020 to 2021. This approach minimizes the risk of selection bias, particularly since our focus was exclusively on native knee joints, thereby enhancing the generalizability of the findings for this population. However, it is important to note that the results are not applicable to prosthetic joints, pediatric populations, or joints other than the knee; thus, no conclusions can be drawn regarding these excluded groups. Limitations of this study includes the risk of informational bias since this is a retrospective study, relying on data from medical records. We did not include non‐culturable pathogens such as B*orrelia burgdorferi*, which may have a different clinical presentation, or 16s‐rRNA PCR sequencing, which makes it a possibility that some cases are falsely negative. Another limitation is that we did not investigate long‐term morbidity, with no data on long‐term sequela for the different diagnoses. Incidences for gout and pyrophosphate arthritis may also be underestimate because they would not always have been subject to arthrocentesis and synovial bacterial culture. We also did not have data on some deaths that occurred outside of the hospital.

## 5 Conclusion

This study found a low incidence of native knee SA in southern Sweden, with gout and pyrophosphate arthritis being twice as common, and observed lower mortality rates than previously reported. Most deaths were non‐preventable and related to comorbidities. These findings highlight the importance of careful diagnostic evaluation in acute knee arthritis, while any implications for antimicrobial use should be interpreted with caution.

## Author Contributions

**Amelia Johansson:** visualization, writing – original draft, investigation, conceptualization, data curation, methodology. **Omid Bornai:** data curation. **Jonas Tverring:** conceptualization, writing – review and editing, methodology, supervision. **Oskar Ljungquist:** conceptualization, writing – review and editing, project administration, methodology, supervision.

## Conflicts of Interest

The authors declare no conflicts of interest.

## Transparency Statement

The lead/Corresponding author (Amelia Johansson) affirms that this manuscript is an honest, accurate, and transparent account of the study being reported; that no important aspects of the study have been omitted; and that any discrepancies from the study as planned (and, if relevant, registered) have been explained. The corresponding author attests that all listed authors meet authorship criteria and that no others meeting the criteria have been omitted.

## Supporting information


Supporting File

## Data Availability

The underlying dataset cannot be shared publicly due to privacy concerns related to the risk of indirect identification. Authors therefore elects to not share the data.
