# Supplementary material for: Septic Arthritis and Its Mimics: Assessing the Incidence, Mortality Rate, Cause‐of Death and Preventability in a Population‐Based Cohort Study
Source: Health Sci Rep. 2026 Aug 6;9(8):e72845. doi: 10.1002/hsr2.72845 (PMC13448159; doi:10.1002/hsr2.72845)
Supplement: Supplementary file 1 [file HSR2-9-e72845-s001.docx]

**SUPPLEMENTAL MATERIAL**

**Table S1.** The predefined study electronic case report form.

**Variable**

Age (years)

Gender (male/female)

Charlson Comorbidity Index (19)

Days of hospital care for the current admission (days)

Rapid Emergency Triage and Treatment System (RETTS, 0-4)

Temperature at ED (degrees, Celsius)

Range of motion (ROM) in affected knee (degrees)

Redness of the knee (yes/no)

Increased warmth of knee compared to the other knee (yes/no),

Joint pain (yes/no)

Unable to bear weight (yes/no)

Suspected skin infection (yes/no).

Intraarticular injection in the joint within 14 days (yes/no)

Knee trauma of the affected knee within 14 days (yes/no)

Previous antibiotic treatment within 14 days (yes/no)

Previous episode of septic arthritis (yes/no)

Previous surgery of the affected knee within 2 months (yes/no)

Number of joints affected

Date of intraarticular injection and

Urine catheterization (yes/no)

Surgery of the affected knee during hospital care (yes/no)

Joint drainage of the affected knee during hospital care (yes/no)

Drug or alcohol abuse (yes/no)

Current or previous history of smoking (yes/no)

Rheumatoid arthritis (yes/no)

Final diagnosis (ICD-10)

Date of death

White blood cells in joint (10^9^/L)

White blood cells in serum (10^9^/L)

Serum C-reactive protein (mg/L)

Erythrocyte sediment reaction (mm)

Serum procalcitonin (µg/L)

Joint glucose (mmol/L)

Serum glucose (mmol/L)

Glucose quotient (glucose ratio between glucose in joint and glucose in serum)

Appearance of the synovial fluid

Crystals in joint (yes/no)

Empirical antibiotics (yes/no)

Start date of antibiotic treatment

Date of discontinued antibiotic treatment

Body mass index (kg/m^2^)

Immunosuppressive therapy (yes/no)

Positive joint cultivation (yes/no)

Cultured bacteria from joint

Positive blood cultivation (yes/no)

Cultured bacteria from blood

Occurrence of arthrosis (yes/no)

**Table S2.** Bacteria considered contaminants.

*Achromobacter* spp.

*Acinetobacter* spp.

*Bacillus* spp.

*Corynebacterium* spp.

*Cutibacterium* spp.

*Dermabacter* spp.

Difteroid rods

Skin flora

*Micrococcus* spp.

*Propionibacterium* spp.

*Rhizobium* spp.

Coagulase Negative Staphylococcus (not including *Staphylococcus lugdunensis*)

**Table S3.** Full list of diagnoses.

| **Diagnoses** | **Number of cultures, n** | **Number of patients, n** |
| --- | --- | --- |
| Arthrosis | 80 | 77 |
| Arthritis/Joint effusion of unknown origin | 119 | 103 |
| Borrelia arthritis | 4 | 4 |
| Bursitis | 18 | 18 |
| By clinicians diagnosed septic arthritis, but when reviewed by two independent researchers not qualified as septic arthritis | 16 | 16 |
| Gout | 126 | 121 |
| Knee pain of unknown origin | 36 | 36 |
| Psoriasis arthritis | 9 | 9 |
| Pyrophosphate arthritis | 139 | 132 |
| Reactive arthritis | 30 | 28 |
| Rheumatoid arthritis | 6 | 6 |
| Ruptures Baker's cyst | 8 | 8 |
| Septic arthritis | 60 | 52 |
| Skin soft tissue infection | 17 | 17 |
| Trauma/contusion | 22 | 22 |
| Acute myeloid leukaemia | 1 | 1 |
| Behcet's disease | 1 | 1 |
| Degenerative stress injury | 1 | 1 |
| Deep vein thrombosis | 3 | 3 |
| Hip fracture | 1 | 1 |
| Intra-articular fracture | 1 | 1 |
| Neurofibromatosis type 1 | 1 | 1 |
| Patella dislocation | 1 | 1 |
| Patella tendinitis | 1 | 1 |
| Pauciarticular juvenile arthritis | 1 | 1 |
| Quadriceps rupture | 1 | 1 |
| Rhabdomyolysis | 1 | 1 |
| Sweet's syndrome | 1 | 1 |
| Injury to knee | 1 | 1 |
| Urinary tract infection | 1 | 1 |
| Viruses | 1 | 1 |
| Unknown infection | 1 | 1 |

**Table S4.** Outcome episodes of septic arthritis, pyrophosphate arthritis and gout arthritis.

| **Outcome** | **Septic arthritis**  *n* = 60 | **Pyrophosphate arthritis**  *n* = 139 | **Gout**  *n* = 126 | **Other diagnoses**  *n* = 384 |
| --- | --- | --- | --- | --- |
| Hospital stay duration, days (median), lower quartile; upper quartile | 16 (9; 22) | 2 (0; 5) | 0 ( 0; 5) | 0 (0; 4) |
| Joint surgery* (%) | 17 (28) | 0 (0) | 0 (0) | 10 (3) |
| Joint rinse (%) | 28 (47) | 4 (3) | 1 (1) | 10 (3) |
| Empiric oral antibiotics (%) | 4 (7) | 1 (1) | 6 (5) | 23 (6) |
| Empiric intravenous antibiotics (%) | 56 (93) | 66 (47) | 41 (33) | 116 (30) |
| Intraarticular corticosteroids (%) | 11 (18) | 44 (32) | 31 (25) | NA |
| Oral corticosteroids (%) | 4 (7) | 52 (37) | 67 (53) | NA |
| Death within 90 days (%) | 5 (8) | 5 (4) | 5 (4) | 5 (1) |
| Death within 30 days (%) | 2 (3) | 1 (1) | 2 (2) | 3 (1) |

*7 episodes obtained arthroscopic joint rinse and synovectomy during the same surgery. 4 episodes received arthroscopic synovectomy after joint rinse. 2 episodes did not get their joints rinsed but instead went through synovectomy direct. 2 episodes underwent arthrotomy and another 2 episodes had to go through fasciotomy and soft tissue incision.
